# Effects of Recreational Ski Mountaineering on Cumulative Muscle Fatigue – A Longitudinal Trial

**DOI:** 10.3389/fphys.2018.01687

**Published:** 2018-11-27

**Authors:** Simon Haslinger, Cornelia Blank, David Morawetz, Arnold Koller, Tobias Dünnwald, Sarah Berger, Nico Schlickum, Wolfgang Schobersberger

**Affiliations:** ^1^1Institute for Sports Medicine, Alpine Medicine and Health Tourism, University for Health Sciences, Medical Informatics and Technology, Tirol, Austria; ^2^Institute for Sports Medicine, Alpine Medicine and Health Tourism, Tirol Kliniken GmbH, Tirol, Austria

**Keywords:** ski mountaineering, muscle fatigue, strength, eccentric, concentric, recreational

## Abstract

Sport is known to have many positive effects on mental and physical health. High-intensity exercise is considered to decrease muscle strength and induce muscle fatigue, which is associated with a higher risk of injury. In recreational alpine skiers, a decrease of eccentric peak hamstring torque, as an indication of muscle fatigue, occurs even after 1 day of skiing. The popularity of ski mountaineering is increasing enormously, but no studies are available on its effects on muscle strength. Therefore, the present study examined the consequences of ski mountaineering on muscle fatigue of the concentric/eccentric quadriceps and/or hamstrings. In addition, a possible role of myofascial foam rolling in reducing muscle fatigue was evaluated. Fifty recreational ski mountaineers (27 males, 23 females) completed five consecutive tours of ski mountaineering within 1 week. After each day of skiing, participants underwent an isokinetic muscle test assessing the concentric and eccentric muscle strength of both thighs. One group completed an additional session of myofascial foam rolling. Right and left concentric quadriceps peak torque, left hamstrings peak torque, left eccentric quadriceps peak torque, as well as right and left hamstring peak torque, were reduced after a single day of ski mountaineering (*p* ≤ 0.016 for all). However, no cumulative muscle fatigue was detected and we could not demonstrate any effect of myofascial foam rolling. The results show conclusively that a single day of ski mountaineering leads to a significant decrease of concentric and eccentric quadriceps and hamstring strength. Therefore, in order to improve muscle strength for the ski mountaineering season, a physical training program including concentric and eccentric methods can be recommended.

## Introduction

Sport is considered to have a positive effect on physical well-being (Amlani and Munir, 2014). Regular physical training is determined to be cardio-protective and preventative against a variety of chronic diseases (Bassuk and Manson, 2005; Powers et al., 2008; Rengo et al., 2013). Additionally, regular physical training has beneficial effects on metabolic syndrome by decreasing the adverse effects of its multifactorial and progressive pathogenesis (Church, 2011). There is also evidence that moderate to vigorous physical activity reduces mortality (by 22%) and hence is recommended for older adults (Hupin et al., 2015). Beyond this, there is some evidence that outdoor physical activity exerts additional positive effects on mental well-being (Thompson Coon et al., 2011).

In contrast, negative effects of physical activity might occur due to the risk of musculoskeletal injuries. In Austria, about 200,000 sports accidents were recorded during 2016. Injuries were most frequently detected in soccer (49,600), alpine skiing and team sports (23,100 and 21,900, respectively) (Kuratorium für Verkehrssicherheit, 2016). Of all injuries recorded (i.e., not only in Austria), the most common are knee and ankle injuries (Habelt et al., 2011), with knee injuries being predominant in alpine skiing (Burtscher et al., 2009; Sulheim et al., 2011; Kim et al., 2012).

Reasons for an increased injury risk may include the well-documented decrease of body stability and balance, as well as proprioceptive factors, during acute physical activity (Chappell et al., 2005; Simoneau et al., 2006; Hassanlouei et al., 2012; Paillard, 2012). Additionally, different types of sports are associated with muscle fatigue, and thus also with an increased risk of injury (Gabbett, 2000; Hiemstra et al., 2001). In general, there are several underlying mechanisms leading to exercise-induced muscle fatigue (e.g., neural, metabolic, oxygen consumption). Nevertheless, all mechanisms ultimately result in a decrease of muscle strength (Wan et al., 2017). Consequently, muscle fatigue is defined as an exercise-induced reduction in the ability of a muscle to generate force or power. It occurs progressively and gradually after the onset of a persistent physical activity (Gandevia, 2001; Enoka and Duchateau, 2008). For example, a 4-h alpine skiing session with normally trained recreational volunteers was associated with a decrease in eccentric quadriceps and hamstring strength, both indicators of muscle fatigue (Koller et al., 2015). In particular, quadriceps and hamstrings are considered to increase knee stabilization and thus protect the anterior crucial ligament. Consequently, reducing muscle fatigue might be important in preventing fatigue-related injury. One possibility for relieving muscle fatigue could be to enhance regeneration and increase flexibility between days of physical activity. In this context, myofascial foam rolling was reported to increase the mobility of fascia and hence to gain possible benefits on performance by increasing the range of motion and ultimately improving recovery. Different approaches concerning the effect of myofascial foam rolling exist. In general, mechanical and neurological aspects are discussed in the current literature. As fascia surrounds each muscle, it is supposed to be involved in the biomechanics of the musculoskeletal system and hence may also be involved in force transmission. Mechanical mechanisms are mentioned to include piezoelectricity, fascial adhesions, cellular responses, fluid flow and fascial inflammation. These mechanisms are supposed to tighten the muscles and fascia. Myofascial foam rolling thereby is assumed to relieve stiffness of fascia and to reduce inflammation by increasing the blood flow. Neurological mechanisms, however, are characterized by involving the Golgi receptors. It is assumed that the pressure that occurs during myofascial foam rolling stimulates these receptors and reduces the motor unit firing rate and thereby decreases muscle tension (Beardsley and Skarabot, 2015; Cheatham et al., 2015; Pearcey et al., 2015; Madoni et al., 2018).

Although evidence regarding injury risk, and potential causes thereof, in different sports exists, there is currently little research in the field of ski mountaineering. However, ski mountaineering is gaining more and more popularity as a leisure winter sports activity, especially in the European Alps (Praz et al., 2016). To the best of our knowledge, no studies have evaluated the effect of ski mountaineering on muscle fatigue thus far, in contrast to the existing evidence regarding alpine skiing (Seifert et al., 2009; Koller et al., 2015). However, whereas during alpine skiing, descending physical activity alternates with passive ascents (by cable car or chair lift) back to the top (Seifert et al., 2009), ski mountaineering consists of both active ascending and descending and therefore may be even more demanding. Furthermore, alpine skiing is characterized by the dominance of eccentric contractions (Berg and Eiken, 1999), whereas ski mountaineering consists of a two-phase activity dominated by concentric contractions in the ascent phase and eccentric contractions during descent (Faulhaber et al., 2007). Hence, it could be expected to produce a more concentric decrease of strength, as ski mountaineering consists of a prolonged uphill phase and only a single descent phase.

Thus, the first aim of this trial was to examine whether ski mountaineering (performed once and repeatedly) leads to muscle fatigue, expressed as decreased muscle strength of the concentric and/or eccentric quadriceps, and/or hamstrings. A second aim was to evaluate if myofascial foam rolling as a regenerative method can reduce muscle fatigue.

We first hypothesized that a single physical load of ski mountaineering decreases muscle strength of the concentric and/or eccentric quadriceps, and/or hamstrings. We additionally hypothesized that a physical load of five consecutive Ski Tours within 1 week would decrease the muscle strength of the concentric and/or eccentric quadriceps, and/or hamstrings, according to cumulative muscle fatigue. Finally, we hypothesized that myofascial foam rolling would decrease the extent of muscle fatigue by counteracting the reduction of muscle strength of the quadriceps and/or the hamstrings. These guiding questions were defined because most recreational ski mountaineers in the Austrian Alps spend on average an entire week of their vacation and thus, there is need of scientific based sports-related recommendations for this specific target group.

## Materials and Methods

### Study Design and Participants

Experienced recreational ski mountaineers were recruited via digital and print media adverts, in local newspapers in Tyrol, Salzburg and South Tyrol. Inclusion criteria were being aged between 40 and 65 years, having an above average skiing level based on a score between 6 and 9 on a visual analog scale (VAS) for skiing (0: “no skiing skills” to 10 “excellent skiing under all terrain and snow conditions”), a minimum of uphill climbing (vertical distance) with touring skis of 8000 m over the time period of the last 2 years, time available for a preliminary examination and a 1-week recreational ski mountaineering holiday, an exercise capacity of more than 110% (age-predicted) based on a cycle ergometry (Lode B.V., Groningen, Netherlands; for details, refer to Koller et al., 2015) and medical approval based on the preliminary examination. Exclusion criteria were acute illnesses and injuries shortly before and during the investigation, a history of knee injuries (i.e., cruciate ligament injuries), chronic diseases, and pain therapy with non-steroidal anti-inflammatory drugs (NSAIDs), corticosteroids and other inflammation inhibitors. Before participants were finally included in the study, they had to provide a signed informed consent form. The study was performed in accordance with the declaration of Helsinki and was approved by the Ethics Committee of the Medical University Innsbruck (AN2016-0209 367/4.4).

In total, 197 persons met the first-line inclusion criteria (pertaining to age, availability, and ski skills) and were subsequently provided with supplementary documents including an information letter, the VAS and a health questionnaire (to obtain data on medical problems and obtain further medical details). Based on the results, 89 persons were invited for medical examinations including medical history, 12-lead electrocardiogram at rest, spirometry, blood sampling (standard laboratory parameters including red and white blood cell counts, thyroid hormones, blood lipids, inflammation values and liver and kidney values), and a maximum cycle ergometry. The most frequent causes for exclusion were pre-existing cardiovascular diseases, such as hypertension or coronary artery disease, chronic obstructive pulmonary disease or another disease requiring treatment, or lack of fitness. In total, 50 volunteers were included (23 females, 27 males), all of whom had to undergo isokinetic dynamometry to gain familiarity with the device, the motion sequence and the testing procedure for the concentric and eccentric muscle strength testing of the quadriceps and hamstrings. The preliminary examinations were conducted at 783 m above sea level.

### Measures

#### Muscle Strength (Isokinetic Dynamometry)

To determine the concentric and eccentric muscle strength of the quadriceps and hamstrings, isokinetic dynamometry was performed using a HUMAC NORM Testing and Rehabilitation System with Evaluation Software HUM6A770 (Proxomed, Ltd., Germany). After warming up for 10 min on a bicycle ergometer (60 Watts females, 80 Watts males), participants were seated on the dynamometer with their hip flexed to approximately 90° and their trunk secured with dual cross-over straps and a waist strap. The range of motion at the knee was set between 0° (fully extended) and 100° (flexed). A thigh strap on the leg to be tested was used to fix the thigh, allowing only flexion and extension. The testing protocol consisted of concentric and eccentric quadriceps and hamstring contractions (four repetitions each at an angular velocity of 60°/s) for familiarization with the equipment, followed by a 15 s brake and then by four maximum repetitions at an angular velocity of 60°/s (Koller et al., 2015). Both legs were tested in each participant.

#### Bioelectrical Impedance Analysis (BIA)

Bioelectrical impedance analysis (Phase-Sensitive Impedance Analyser BIA 101, Akern s.r.l., Florence, Italy), as a non-invasive method to measure body composition, was performed to monitor fluid balance during the vacation (Bosy-Westphal et al., 2006).

#### Heart Rate Monitoring and GPS-Tracking

A sports watch (Suunto Ambit2 R, Finland) was used to store and monitor heart rate (HR), ascent time, ascent altitude difference, ascent distance and GPS tracking data to ensure a similar workload to all subjects. Participants were grouped based on their exercise capacity.

#### Myofascial Foam Rolling (MFR)

Half of the participants performed a self-myofascial release to the muscle groups of the lower extremity and the buttocks using the Black Roll^®^. Training was performed under the supervision of a qualified sports scientist, following a previously used protocol (Pearcey et al., 2015).

#### Questionnaires

To avoid participants attending the study while suffering from health issues, their mental and physical state was assessed with a VAS (-5 = very bad, 0 = neutral, +5 = very good). Additionally, the 15-point Borg scale to assess perceived exhaustion (range: 6 to 20; 6 = none, 9 = very easy, 20 = maximum) was applied (Borg, 2004). The Scale was only used to assess if the participants exercised with a comparable subjective exercise intensity. For the analyses the Borg scale was further classified into three sub-groups: 1 = light exhaustion (Borg values 6–11), 2 = moderate exhaustion (Borg values 12–14) and 3 = heavy exhaustion (Borg values 15–20). The VAS was filled in before each Ski Tour, and the BORG-15-point scale was filled in after the Ski Tour but before the isokinetic dynamometry.

Additionally, two questionnaires were included to assess whether objective indicators of muscle fatigue and potential stress can be aligned to the subjective perception of well-being. To obtain the stress reactivity potential as a state of current strain and recovery, the shortened German Version (EBF 24 B/3) of the Recovery-Strain Questionnaire (RESTQ) was used (Kellmann and Golenia, 2003; Kellmann, 2010). The questionnaire covers seven different subtests of strain (general-, emotional-, social- and somatic strain, conflict, fatigue, and lack of energy) as well as five subtests of recovery (performance, sleep, social-, somatic-, and general- recovery) (Kallus, 2011). The individual scales have a score range between 1 and 6, with lower values indicating a low expression of the specific scale.

In addition, the German “Befindlichkeitsskala” (Feeling Scale), a multidimensional method to obtain the current mood state, was included. It is based on the bipolar basic dimension of “tension” and “assessment” (Abele-Brehm and Brehm, 1986). The shortened German Version of the Bf-SR, consisting of a 24 items list to assess the current psycho-physical state through contrary adjectives (i.e., undecided, decisive, neither-nor), was used (Von Zerssen and Petermann, 2011). The score of the scale has a range between 0 and 48, with lower values indicating a more positive mood state.

### Procedure

The trial took place at the ski area of the village of St. Johann (659 m above sea level), Tyrol, Austria during 2 weeks in March 2017. Participants performed five ascents and descents within 6 days, including a rest day (day 4). Baseline fluid balance and strength values were assessed on the day of arrival. Participants were subsequently assigned to matched intervention I_F_ (Foam roll = active regeneration with myofascial foam roll) and I_NF_ (No Foam roll = no active regeneration) groups based on age, exercise capacity and muscle strength. Additionally, participants were grouped into performance categories (low, middle, high) based on their respective exercise capacity (Watt_max_ per kilogram body weight and maximum heart rate) after incremental maximal cycle ergometry. Groups were advised to ascend the mountains with a heart rate resembling a submaximal exercise of 70–85% of their maximal heart rate. Thus, the individual cardiopulmonary strain during ski mountaineering should be comparable among all volunteers. Furthermore, all participants were instructed about the routes and safety aspects of ski mountaineering by certified mountain guides (UIAGM). During the vacation, alcohol consumption was limited to an amount equal to one glass of beer (500 ml) per day, to avoid any negative influence on the fluid balance.

The ski mountaineering trials were all accomplished in the morning to ensure similar conditions during the 2-week investigation. The first Ski Tour was planned to be shorter to serve as a day of accustoming to the environmental conditions and to the security equipment. Furthermore, it was necessary for the guides to visualize and assess participant’s technical skills for the further planning. Participants were instructed to keep up with the pace corresponding to their baseline endurance capacity. The mountain guides accompanying each group were also instructed to monitor the predefined exercise load according to distance, pace and ascent. All groups completed the same course, including an ascent of 2 to 3 h, depending on the vertical height of the summit, with an overall duration of 5 h including breaks. Guides were responsible to command the simultaneous start and stop of the sport watches at the beginning and at the end of each Ski Tour. Within 1–3 h post-exercise, participants underwent the isokinetic dynamometry following a 10-min warm up on an indoor cycle. Participants of group I_F_ additionally underwent supervised myofascial foam rolling training. Upon completion of the test procedure, participants could relax and were allowed to use the sauna. Performance of active regeneration methods, such as different types of work outs or massages, was not permitted.

Passive regeneration was planned for the rest day and active regeneration was prohibited except a leisurely walk (e.g., sightseeing). BIA was performed to monitor fluid balance.

**FIGURE 1:**
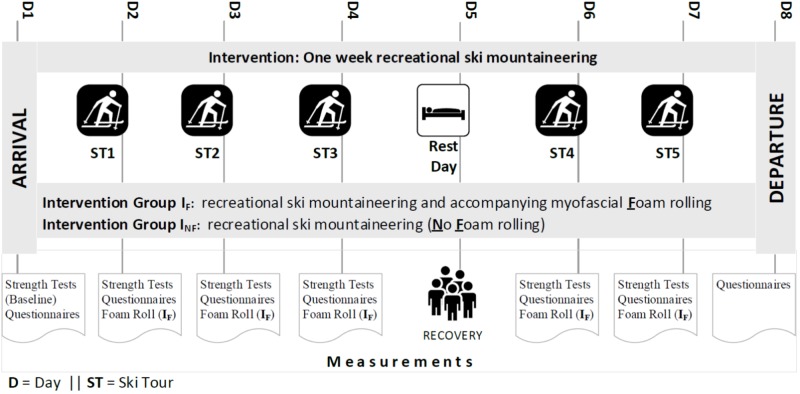
Study procedure.

Questionnaires were handed out to the participants three times – at the study inclusion (baseline, to verify equal distribution between I_F_ and I_NF_ group), at the arrival- and departure day. Details of the procedure are outlined in Figure 1.

### Statistical Analysis

SPSS 24.0 was used to perform the statistical analyses. Data showed a normal distribution on graphical evaluation. Numerical data are presented as mean ± SD. To confirm successful group matching (foam roll vs. no foam roll), *t*-tests (metric data) and chi-square tests (nominal and ordinal data) were applied. To confirm a successful distribution of the participants within the performance groups, a univariate analysis of variance (ANOVA) or Welch-test (depending on variance) was applied for the interval-scaled variables (HR, time, ascent, and distance) and a Chi-square test was applied for the Borg values. In case of significance, Bonferroni or Tamhane-corrected *post hoc* tests were applied. The effects of a single ski mountaineering tour on muscle fatigue were assessed with a 3 × 2 mixed linear model initially including performance group as fixed factor to control for a potential effect of the grouping on changes in muscle strength. In case of no significant interaction effect, a paired *t*-test was performed to assess the effect of a single ski mountaineering tour on muscle fatigue. Cumulative effects of repeated Ski Tours on strength as well as a potential regenerative effect of the foam roll were analyzed using a 3 × 2 × 4 linear mixed effect model on repeated measures including the performance group effect, a strength effect, a comparison group effect (foam roll), as well as possible interaction effects. In case of no significant effect of the performance group on the development of muscle strength over time, a simplified 2 × 4 linear mixed model was applied. In cases of significant interaction effects (strength × foam roll group), groups were analyzed separately. Effects of Ski Tours on well-being were analyzed using a linear mixed effect model on repeated measures including a well-being effect as well as a comparison effect (well-being × foam roll group). In case of significant interaction effects, well-being was analyzed for the two comparison groups separately. For strength and well-being effects, Sidak corrected *post hoc* tests were applied. The level of significance was set at *p* < 0.05, and effect sizes were provided as Cohens d and partial eta square (η^2^).

## Results

### Study Sample

In total, 50 recreational ski mountaineers, 23 females (age 48.3 ± 4.4 years, height 168.1 ± 6.6 cm, weight 59.5 ± 6.5 kg) and 27 males (age 51.4 ± 6.8 years, height 178.4 ± 7.0 cm, weight 76.0 ± 10.2 kg), were included in this study. Baseline values of peak torque (N m) were examined in both genders: concentric muscle functioning (left quadriceps: 143.2 ± 45.0, right quadriceps: 144.6 ± 46.2; left hamstrings: 94.8 ± 27.1, right hamstrings: 92.6 ± 25.6) and eccentric muscle functioning (left quadriceps: 200.92 ± 64.2; right quadriceps: 189.7 ± 48.7; left hamstrings: 122.0 ± 36.7, right hamstrings: 120.0 ± 29.4). Gender distribution, age, and maximum power according to weight and muscle strength (peak torque) of the quadriceps and hamstrings (concentric and eccentric flexion and extension), did not significantly differ between I_F_ and I_NF_ group (*p* > 0.15 to *p* < 0.99 for all). Additional evaluation showed a right-leg dominance in most subjects (91.8%). Furthermore, only 37% of the participants accepted the sauna offer, the frequency of sauna sessions was similar in both groups (*p* = 0.64). Fluid balance remained unchanged during the study week (*p* = 0.32). One subject was excluded after the rest day due to painful ankle pressure caused by the ski boot. Thus, 49 participants finished the trial.

No significant differences in HR, ascent time, distance and or altitude difference were observed between groups I_NF_ and I_F_ (*p* ≥ 0.11). The prior grouping into three performance groups were successful as it showed no significant differences in HR, except for the first day of ski mountaineering, as it was a day of adaption to the environmental conditions. The overview of the perceived exhaustion by using the Borg scale, did not exhibit significant differences either (for further information refer to Table 1). The rate of perceived exhaustion showed a “light” to “moderate” exhaustion and remained constant throughout all five tours of ski mountaineering. No significant differences, between either the I_NF_ and I_F_ groups (*p* = 0.186), or between the performance groups (*p* ≥ 0.103), were observed during the 5 days of physical activity. Thus, a similar and moderate exercise load was confirmed for all participants. The average load carried was 16.4 ± 2.5 kg, calculated as the difference in bodyweight, with versus without equipment (backpack, skis, and ski poles), measured immediately before the first Ski Tour.

**Table 1:** Descriptive data regarding the ski route, heart rate, and perceived exhaustion of the recreational ski mountaineers during 5 days of ski mountaineering [Ski Tour 1 (ST1) to Ski Tour 5 (ST5)].

		ST1	ST2	ST3	ST4	ST5
	Group	(Mean ±*SD*)	(Mean ±*SD*)	(Mean ±*SD*)	(Mean ±*SD*)	(Mean ±*SD*)
Time (min)	High	94 2*	185 11#	157 6*#	125 7*	149 7*
	Middle	95 1*	176 4*	170 19	128 8*	150 11*
	Low	140 0	192 1	175 14	166 1	168 8
HR (bpm)	High	158 10*	133 11	134 10	139 11	129 9
	Middle	153 16	136 14	135 13	138 11	129 9
	Low	141 15	131 13	129 13	135 14	130 13
Ascent (m)	High	949 10	1134 19*#	1203 21*	1139 162	976 40
	Middle	947 10	1085 25*	1162 124*	1181 180	984 37
	Low	952 3	974 45	966 24	1225 298	976 47
Distance	High	4390 218*	6655 5	5600 934*	4545 835*	5645 566
	Middle	4217 36*	6612 134	5602 484*	4600 820*	5878 436
	Low	4968 323	6496 407	6644 1000	5914 959	5907 549
		**ST1** (Median IQR)	**ST2** (Median IQR)	**ST3** (Median IQR)	**ST4** (Median IQR)	**ST5** (Median IQR)
Borg	High	2.00 0.25	2.00 1.00	2.00 1.00	2.00 1.00	2.00 1.00
	Middle	2.00 1.00	2.00 1.00	2.00 1.00	1.00 1.00	1.00 1.00
	Low	2.00 1.00	1.00 1.00	2.00 1.00	2.00 1.00	2.00 1.00


*Time = ascent time, HR = average heart rate during ascent, Ascent = ascent altitude difference, Distance = ascent distance. ^#^*p* < 0.05 compared to “middle,” ^∗^*p* < 0.05 compared to “low.”*

### Effects of a Single Day of Ski Mountaineering on Muscle Fatigue

Performance group did not show a significant effect on the development of any parameter of muscle strength over the two time points (*p* > 0.05). Results of the paired *t*-test indicated that concentric quadriceps peak torque (left and right) and hamstrings peak torque (left), as well as eccentric hamstrings peak torque (left and right) and quadriceps peak torque (left), showed a significant reduction after the first day of ski mountaineering (ST1) compared to baseline on arrival day (AD) (*p* ≤ 0.018 for all). Details are outlined in Table 2.

**Table 2:** Concentric and eccentric isokinetic strength test of the recreational ski mountaineers, on the arrival day and after the first day of ski mountaineering (ST1).

	Concentric					Eccentric				
Muscle	*n*	Arrival	ST1	*p*	*d*	*n*	Arrival	ST1	*p*	*d*
QUAD__left_	50	143.2 ± 45.0	136.8 ± 44.3	0.009	0.39	50	200.92 64.2	190.8 66.1	0.016	0.36
QUAD__right_	50	144.6 ± 46.2	139.4 ± 44.6	0.013	0.36	49	189.7 48.7	183.7 47.3	0.166	0.20
HAM__left_	50	94.8 ± 27.1	90.7 ± 27.5	0.015	0.36	50	122.0 36.7	113.1 33.4	< 0.001	0.57
HAM__right_	50	92.6 ± 25.6	93.8 ± 26.4	0.234	0.17	49	120.0 29.4	115.2 27.6	0.018	0.34


*QUAD = quadriceps, HAM = hamstrings. Paired *t*-test was conducted. Significant difference (*p* < 0.05). Values are shown as mean ± SD.*

### Effects of 5 days of Skiing on Cumulative Muscle Fatigue

#### Concentric Peak Torque

Performance group did not show a significant effect on the decrease of muscle strength over time except for one parameter (interaction effect performance group × muscle strength: *p* = 0.041). There was a significant increase in quadriceps peak torque in the right leg but only in performance group one (the fastest group) (*p* = 0.002). In detail, muscle strength significantly increased comparing ST4 and ST5 (*p* = 0.011). For all other variables, the simplified 2 × 4 mixed linear model was used for the remaining parameters.

No cumulative muscle fatigue effects, i.e., a decrease in muscle strength for the quadriceps and hamstrings muscles of either thigh, were detected at four measurement time points (ST1-ST3-ST4-ST5). However, the data revealed a significant increase in hamstrings peak torque of the right leg (*p* = 0.001), with an effect on strength over time (ST1-ST5, *p* = 0.046; and ST3-ST5, *p* = 0.006). As there was a rest day on day 4, additional analysis with four measurement time points were conducted to evaluate the impact of the rest day. No significant changes were found. Details are outlined in Table 3.

**Table 3:** Concentric isokinetic strength test of recreational ski mountaineers during 5 days of ski mountaineering (ST1 to ST5).

								ANOVA (ST1-ST3-ST4-ST5)			
Muscle	G	*n*	ST1	ST2	ST3	ST4	ST5	Strength		Strength × Group	
								*p*	η^2^	*p*	η^2^
QUAD__left_	I_NF_	24	132.8 ± 40.0	133.3 ± 38.3	130.1 ± 40.6	133.1 ± 42.5	129.7 ± 42.0	0.973	0.002	0.285	0.026
	I_F_	25	138.6 ± 48.5	139.0 ± 45.1	142.3 ± 46.7	139.4 ± 41.7	144.4 ± 46.1				
HAM__left_	I_NF_	24	86.7 ± 21.0	89.0 ± 20.7	87.3 ± 23.5	88.2 ± 22.9	87.4 ± 23.8	0.068	0.049	0.173	0.035
	I_F_	25	94.8 ± 33.0	96.0 ± 29.6	98.6 ± 28.8	99.4 ± 25.5	103.0 ± 31.2				
HAM__right_	I_NF_	24	88.8 ± 22.9	87.7 ± 21.1	88.3 ± 25.2	91.6 ± 23.1	93.5 ± 25.4	0.001	0.116	0.939	0.003
	I_F_	24	98.2 ± 30.2	97.1 ± 28.9	99.3 ± 31.1	102.0 ± 30.4	103.4 ± 28.5				


*G = group, QUAD = quadriceps, HAM = hamstrings. ANOVA (four measurement time points) was conducted, Significant difference (*p* < 0.05). Values are shown as mean ± SD.*

#### Eccentric Peak Torque

Performance group did not show a significant effect on the decrease of muscle strength over time except for one parameter (interaction effect performance group × muscle strength: *p* = 0.017). There was a significant increase in quadriceps peak torque in the right leg but only in performance group one (the fastest group) (*p* = 0.006). In detail, muscle strength significantly increased comparing ST1 and ST5 (*p* = 0.036) and ST4 and ST5 (*p* = 0.047). For all other variables, the simplified 2 × 4 mixed linear model was used for the remaining parameters.

No significant decrease of peak torque, of the hamstrings of either thigh or the left quadriceps, was detected at any of the four measurement time points (ST1-ST3-ST4-ST5). Details are provided in Table 4.

**Table 4:** Eccentric isokinetic strength test of recreational ski mountaineers, during 5 days of ski mountaineering (ST1 to ST5).

								ANOVA (ST1-ST3-ST4-ST5)			
Muscle	*G*	*n*	ST1	ST2	ST3	ST4	ST5	Strength		Strength × Group	
								*p*	η^2^	*p*	η^2^
QUAD__left_	I_NF_	24	179.9 ± 47.4	183.0 ± 50.6	183.6 ± 50.5	189.6 ± 60.4	178.8 ± 60.3	0.725	0.009	0.393	0.021
	I_F_	25	197.2 ± 78.6	206.8 ± 69.1	208.3 ± 83.9	196.8 ± 53.1	207.0 ± 63.7				
HAM__left_	I_NF_	24	107.2 ± 26.7	111.7 ± 25.1	109.0 ± 28.7	110.3 ± 28.7	108.9 ± 28.0	0.372	0.022	0.523	0.016
	I_F_	24	114.0 ± 30.6	114.1 ± 30.2	110.3 ± 27.0	116.1 ± 34.3	116.7 ± 30.2				
HAM__right_	I_NF_	24	114.8 ± 26.1	109.8 ± 25.8	108.8 ± 29.2	114.3 ± 31.3	109.4 ± 31.3	0.317	0.025	0.155	0.037
	I_F_	23	113.9 ± 30.0	114.1 ± 28.5	113.5 ± 32.5	115.6 ± 31.1	119.7 ± 31.0				


*G = group, QUAD = quadriceps, HAM = hamstrings. ANOVA (four measurement time points) was conducted, Significant difference (*p* < 0.05). Values are shown as mean ± SD.*

### Effects of Myofascial Foam Rolling on Muscle Fatigue

Also after controlling for a possible effect of performance group, no interaction effects were found for any of the muscle strength (details in Tables 3, 4) and mood indicators.

### Mood and Well-Being

Overall, values of mood and strain scales of well-being were rather low indicating a positive mood and low strain (for details refer to Table 5). Mood has significantly improved in both groups with a medium effect (η^2^ = 0.13, *p* = 0.02).

**Table 5:** Subjective mood and well-being.

	*G*	*n*	Arrival	Departure	ANOVA			
					Time		Time × Group	
					*p*	η^2^	*p*	η^2^
Mood	I_NF_	24	7.05 ± 7.4	4.16 ± 3.8	0.002	0.13	0.643	0.006
	I_F_	25	5.29 ± 5.5	3.33 ± 2.8				
General strain	I_NF_	24	0.65 ± 0.9	0.23 ± 0.4	<0.001	0.26	0.991	<0.001
	I_F_	25	0.66 ± 0.6	0.22 ± 0.3				
Emotional strain	I_NF_	24	0.56 ± 0.7	0.38 ± 0.4	0.014	0.12	0.754	0.002
	I_F_	25	0.68 ± 04	0.44 ± 0.5				
Social strain	I_NF_	24	0.89 ± 0.9	0.67 ± 0.5	0.003	0.18	0.166	0.04
	I_F_	25	1.1 ± 0.7	0.56 ± 0.6				
Conflicts	I_NF_	24	1.13 ± 0.9	0.94 ± 0.7	0.461	0.01	0.033	0.09
	I_F_	25	1.10 ± 0.9	1.5 ± 1.0				
Fatigue	I_NF_	24	1.15 ± 0.9	0.90 ± 0.7	0.004	0.16	0.374	0.02
	I_F_	25	1.34 ± 1.1	0.88 ± 0.8				
Lack of energy	I_NF_	24	0.92 ± 0.7	0.75 ± 0.6	0.196	0.04	0.832	0.001
	I_F_	25	1.02 ± 0.5	0.90 ± 0.6				
Somatic strain	I_NF_	24	0.77 ± 0.8	0.92 ± 0.6	0.242	0.03	0.981	<0.001
	I_F_	25	0.94 ± 0.7	1.08 ± 0.7				
Performance	I_NF_	24	2.78 ± 1.1	1.96 ± 1.4	<0.001	0.36	0.266	0.03
	I_F_	25	3.33 ± 0.8	2.02 ± 1.3				
Social recovery	I_NF_	24	3.02 ± 0.8	4.27 ± 1.1	<0.001	0.41	0.239	0.03
	I_F_	25	3.18 ± 0.7	4.00 ± 1.1				
Somatic recovery	I_NF_	24	4.10 ± 1.1	4.92 ± 0.5	<0.001	0.35	0.03	0.10
	I_F_	25	4.34 ± 0.8	4.64 ± 0.8				
General recovery	I_NF_	24	4.06 ± 1.1	4.83 ± 0.7	<0.001	0.43	0.06	0.07
	I_F_	25	4.48 ± 0.7	4.88 ± 0.7				
Sleep	I_NF_	24	4.67 ± 1.1	4.67 ± 1.0	0.245	0.03	0.245	0.03
	I_F_	25	4.88 ± 0.9	4.62 ± 0.9				


*G = group. ANOVA was conducted. Significant differences (*p* < 0.05). Values are shown as mean ± SD. Lower values indicate a better mood.*

Four of the seven strain scales (general, emotional, social, fatigue) of the well-being questionnaire indicated a significant decrease of strain, one indicated a significant increase of strain but only in the intervention group (conflicts), and no changes were detected in two of the seven scales (lack of energy, somatic strain). Results are detailed in Table 5.

Three out of the five recovery scales indicated significantly higher values at the departure day (social, somatic, general) one of the scales indicated significantly lower values (performance) and one did not significantly change (sleep). Results are detailed in Table 5.

In terms of strain, except for “conflicts” that only increased in the I_F_ group (*p* = 0.03), there were no differences between I_F_ and I_NF_ group. For recovery, results indicate that except for somatic recovery (*p* = 0.03), no interaction effects between the groups were detected. Values indicate that both groups significantly recover from a somatic perspective and that the I_NF_ group’s recovery is significantly stronger. Additionally, we detected a tendency that the I_NF_ group showed a higher recovery also in terms of general recovery (*p* = 0.06) (refer to Table 5).

## Discussion

The aim of this study was to examine if ski mountaineering (single and repeated sessions) is associated with muscle fatigue of the concentric and/or eccentric quadriceps, and/or hamstrings, expressed as a decrease in muscle strength. Additionally, we evaluated the effects of myofascial foam rolling on the recovery from muscle fatigue. We found a significant decrease of concentric and eccentric quadriceps and hamstring strength after a single day of ski mountaineering, but no cumulative decrease of strength during repeated days of ski mountaineering. Myofascial foam rolling showed no effect on recovery.

### Muscle Fatigue After a Single Day of Recreational Ski Mountaineering

In contrast to 4 h of recreational alpine skiing, wherein a decrease in eccentric muscle strength of approximately 10% was observed (Koller et al., 2015), we show significant decreases in the concentric and eccentric function of the involved muscles in both thighs after a single day of ski mountaineering, of up to 5% (concentric) and 8% (eccentric). As ski mountaineering predominately involves a prolonged concentric exercise load during ascent, a loss of concentric quadriceps and hamstring strength is to be expected. An additional reason for significant fatigue after only 1 day might relate to environmental factors, namely snow conditions, present in our study, including deep snow, powder, corn, and crusted snow. In contrast to alpine skiing, which takes place on pre-prepared slopes, the conditions encountered by ski mountaineers are typically more difficult to navigate. Moreover, ski mountaineers usually carry a large amount of equipment, which might have a higher impact during the ascent and the descent. In particular, this could lead to a further decline in both concentric and eccentric strength, which was shown to result in neuromuscular impairment by decreasing voluntary contractions while carrying a backpack (Blacker et al., 2013).

### Cumulative Muscle Fatigue During 1 week of Recreational Ski Mountaineering

Relative to the single ski mountaineering tour, no additional decrease of strength was recorded during 1 week of consecutive recreational ski mountaineering. Nevertheless, despite being mostly non-significant, the descriptive data showed that peak torque values generally remained at a lower level compared to the baseline measurement, with small to medium effect sizes (Tables 2, 3). In our study, the period of recovery was about 16–20 h before the next day of ski mountaineering. Prior research featuring a prolonged session of recreational alpine skiing on prepared slopes reported a reduced eccentric peak torque 24 h after the skiing session (Koller et al., 2015).

Similar results were found by an investigation of consecutive downhill running performed daily for 6 days, in which the maximal isometric contractions remained at a lower level compared to baseline throughout 7 days of measurement (Chen et al., 2008).

Another study on running economy during downhill running showed a decrease in maximal isometric strength, even up to 5 days after the running exercise (Chen et al., 2009). Therefore, a recovery period of 16–20 h might prevent increased muscle fatigue; however, this appears to be insufficient to fully recover from the strength loss. Thus, an additional rest day might increase the degree of recovery. On the contrary, foregoing a rest day during a period of consecutive ski mountaineering might increase muscle fatigue and have a greater effect on cumulative muscle fatigue.

Since during the ski tours our participants were exposed to short periods of moderate hypoxia an influence of repeated hypoxic phases on muscle fatigue needs to be discussed. It is well-known that hypoxia has negative consequences on muscular performance and increases central and peripheral muscle fatigue (Morales-Artacho et al., 2015; Lloyd et al., 2016; Alhammoud et al., 2018). At present it is unclear if acclimatization to hypoxia can at least partially alleviate the reduced muscle contractile properties. Since our volunteers were only exposed to moderate hypoxia for a few hours per day and since they were staying at low altitude before and after exercise, even the total hypoxic exposure time over the whole week may be too short for profound adaptive processes. However, we cannot fully exclude that the missing steady decline of muscle fatigue during the ski touring vacation is partially based upon acclimatization effects.

Analysis of the BORG scale results demonstrated a moderate perceived level of exertion during the study week. Thus, it appears that the targeted and instructed pace of an individual’s submaximal exercise capacity have been met, also concerning the HR during the ascent. An increase of exercise load on the musculoskeletal system (i.e., speed increase, steeper terrain, greater technical demands during skiing and/or climbing) might lead to a more pronounced decrease of strength in 1 day, as well as over the following days during a 1-week skiing holiday, with the potential for accumulated muscle fatigue. In contrast to our expectations, the maximal peak torque of the right hamstrings in its concentric phase, and of the right quadriceps in its eccentric phase, increased significantly during five tours of ski mountaineering (from ST1 to ST5). As almost 92% of the participants were right leg-dominant, we assume that the dominant leg is less vulnerable to fatigue. However, the literature is inconclusive concerning the influence of leg dominance on muscle fatigue. Nevertheless, it is commonly accepted that leg dominance should be considered in the context of specific movements and skills, and may have clinical importance with respect to quantifying asymmetric performance levels in the context of injury, rehabilitation and return to action (McGrath et al., 2016; Van Melick et al., 2017). In addition, a possible learning effect relating to the daily isokinetic dynamometry test might have affected muscular strength and thereby either led to a steady state at a lower level, or even to the aforementioned increase of strength during the course of the week. A prior investigation indicated that acquisition of motor skills during a dynamic movement can improve muscle strength (Connelly et al., 2000). In fact, this could disguise a cumulative loss of strength and thus any accumulation of muscle fatigue might be underestimated in this study. Furthermore, a potential training effect, concerning both the subsequent isokinetic dynamometry as well as the exercise load during consecutive ski mountaineering, might also have influenced the results due to an underestimation of cumulative muscle fatigue. In line, the overall performance scale of the strain-recovery questionnaire was the only one significantly decreasing over the course of the one week of ski mountaineering. Even though participants perceived the level of exertion as light, the responses of the questionnaire indicate a decrease in overall performance possibly supporting the possibility of a subjective underestimation of fatigue.

### The Effect on Recovery of Myofascial Foam Rolling During 1 Week of Recreational Ski Mountaineering

Prior studies showed that MFR may promote recovery after exercise. In this context, a systematic review by Beardsley and Skarabot (2015) assessed the acute and chronic effects of MFR and found a positive effect for athletes and the general population in terms of enhanced recovery from exercise and training. However, our findings did not support this assumption and we found no beneficial effect related to muscle fatigue. We even found detrimental effects from a subjective well-being perspective as results indicated higher somatic recovery and less conflicts in the I_NF_ group compared to the I_F_ group. Previous studies focused on flexibility and joint range of motion, reduction of pain and muscle soreness, whereas our primary outcome was strength loss as a product of muscle fatigue. This difference in outcome variables might be a reason for the absence of any effect of MFR in the current study. However, concerning only the thigh strength, our findings are in accordance with results of a recent study which reported no significant changes in eccentric hamstrings peak torque and a significant decrease of quadriceps peak torque after myofascial foam rolling (Madoni et al., 2018). Furthermore, we used the protocol of Pearcey et al. (2015); however, the effectiveness of different MFR methods are controversial in the literature. In this context, a systematic review indicated an absence of consensus concerning the optimal intervention method (Cheatham et al., 2015). Overall, MFR seems to have a valuable effect on certain outcome measures, but might not affect strength loss *per se*. Hence, it is not justified to reject MFR as method for recovery during 1 week of ski mountaineering in terms of objective physiological values but might be questionable from the perspective of perceived recovery.

### Impact of Muscle Fatigue on Injury Risk

Prior studies reported that muscle fatigue reduced and delayed activation of the quadriceps and hamstring muscles in response to rapid destabilizing perturbations, with a presumably increased risk of injury (Hassanlouei et al., 2012). In particular, eccentric reduction of muscular force had a large impact on injury risk (Armstrong et al., 1983; Lindstedt et al., 2001). Concerning active recreational vacationers, muscle fatigue, as seen in this study after a single day of ski mountaineering, might increase injury risk and thus lead to a premature ending to the holiday.

### Limitations

We cannot exclude a possible learning effect due to the repeated tests using the isokinetic dynamometer. However, no conclusive data exists regarding the number of tests needed to improve exercise performance. Furthermore, the motivation levels of the participants might have affected the results of the strength tests. In contrast to laboratory-based designs, outdoor investigations are always influenced by environmental conditions. Nevertheless as we tried to ensure analogical conditions during the study week, changing snow conditions daily by varying the routes might have influenced the results over the entire week. However, each single day was standardized by means of selecting the same mountain route for all participants. The Borg scale has its limitations for environmental conditions (bad weather), a possible very brief period of high strain (kick turns), and the individual suffering capacity (individual condition of the day).

## Conclusion

We provide evidence that at least only 1 day of ski mountaineering can lead to a significant decrease of concentric and eccentric quadriceps and hamstring strength, indicative of muscle fatigue. Therefore, *a priori* endurance training and additional concentric and eccentric methods to improve strength and prepare for the forthcoming exercise load might be suggested. Furthermore, improving strength and endurance also seems advisable to meet the technical demands of the sport (Hebert-Losier and Holmberg, 2013; Vogt and Hoppeler, 2014). Thus, it might be possible to increase the daily duration of consecutive ski mountaineering sessions under a consistent and moderate exercise load. Conversely a ski mountaineering sessions of a fixed duration could be performed at a higher intensity level. In any case, during consecutive days of ski mountaineering, it might be advisable to introduce a rest day to enhance the likelihood of recovery.

## Author Contributions

SH, CB, WS, AK, DM, and TD contributed to the conception and design of the study. SH, AK, and DM managed the recruitment process and the preliminary examinations. SH, DM, TD, SB, and NS conducted the experiments. SH, DM, and CB organized the database. SH and CB performed the statistical analysis. SH drafted the manuscript. All authors contributed to the manuscript revisions, and read and approved the submitted version.

## Conflict of Interest Statement

The authors declare that the research was conducted in the absence of any commercial or financial relationships that could be construed as a potential conflict of interest.
